# Associations between Diet and Toenail Arsenic Concentration among Pregnant Women in Bangladesh: A Prospective Study

**DOI:** 10.3390/nu9040420

**Published:** 2017-04-23

**Authors:** Pi-I. D. Lin, Sabri Bromage, Md. Golam Mostofa, Joseph Allen, Emily Oken, Molly L. Kile, David C. Christiani

**Affiliations:** 1 Department of Environmental Health, Harvard T.H. Chan School of Public Health, Boston, MA 02113, USA; pil864@mail.harvard.edu (P.-I.D.L.); jgallen@hsph.harvard.edu (J.A.); molly.kile@oregonstate.edu (M.L.K.); 2 Research Center for Environmental Medicine, Kaohsiung Medical University, Kaohsiung 80709, Taiwan; 3 Department of Nutrition, Harvard T.H. Chan School of Public Health, Boston, MA 02113, USA; sbromage@mail.harvard.edu; 4 Department of Environmental Research, Dhaka Community Hospital, Dhaka 1217, Bangladesh; mostofa07@gmail.com; 5 Department of Population Medicine, Harvard Medical School and Harvard Pilgrim Health Care Institute, Boston, MA 02115, USA; emily_oken@harvardpilgrim.org

**Keywords:** food frequency questionnaire, arsenic exposure, pregnancy, Bangladesh, dietary assessment

## Abstract

This prospective study evaluated the relationship between long-term dietary habits and total arsenic (As) concentration in toenail clippings in a cohort of 1616 pregnant women in the Bangladeshi administrative regions of Sirajdikhan and Pabna Sadar. Diet was assessed at Gestation Week 28 and at Postpartum Month 1, using a locally-validated dish-based semi-quantitative food-frequency questionnaire. Toenail As concentration was analyzed by microwave-assisted acid digestion and inductively coupled plasma mass spectrometry. Associations between natural log-transformed consumption of individual food items and temporally matched natural log-transformed toenail As concentration were quantified using general linear models that accounted for As concentration in the primary drinking water source and other potential confounders. The analysis was stratified by As in drinking water (≤50 μg/L versus >50 μg/L) and the time of dietary assessment (Gestation Week 28 versus Postpartum Week 1). Interestingly, toenail As was not significantly associated with consumption of plain rice as hypothesized. However, toenail As was positively associated with consumption of several vegetable, fish and meat items and was negatively associated with consumption of rice, cereal, fruits, and milk based food items. Further studies in pregnant women are needed to compare As metabolism at different levels of As exposure and the interaction between dietary composition and As absorption.

## 1. Introduction

The World Health Organization includes arsenic (As) in its list of 10 chemicals of major public health concern. The many adverse health outcomes linked to As exposure include skin lesions, cancers, and cardiovascular diseases [1]. High concentrations of this human toxicant in ground water have been reported in Argentina, Bangladesh, Chile, China, India, Mexico, and the United States of America. Therefore, As in drinking water is a global health concern that may impact over 200 million people [2,3]. In areas with high water As levels in the ground water, As exposure may occur directly through consumption of As-contaminated drinking water or indirectly through consumption of foods, e.g., agricultural produce and livestock that accumulate As from contaminated water, soil, pesticides, feed, feed supplements, and foraged grasses and plants [4,5,6,7,8,9]. In areas without As contamination, seafood is the main dietary source of arsenic [10]. Arsenic exists in organic and inorganic forms, both of which are readily absorbed (70%–90%) by the gastrointestinal tract. Inorganic forms of arsenic, including the trivalent arsenite (As^III^) and the pentavalent arsenate (As^V^), are more toxic compared to the organic forms, such as arsenobetaine, arsenolipids, and arsenosugars that are mainly found in fish and shellfish [11]. Detailed exposure assessments show that As in food and As in drinking water contribute similarly to the internal dose of As intake [12]. Intake of As from food had often been overlooked due to the complexity of assessing As exposure from diet. Nevertheless, total As ingested by an individual may be underestimated if only drinking water As concentration is considered since As accumulates in food during cultivation and during cooking, especially in areas with high As concentrations in the ground water [9]. 

In Bangladesh, which is an area of endemic high inorganic As (iAs) exposure, accurately estimating dietary exposure to As is particularly important because the iAs concentration in drinking water typically exceeds the standard of 50 μg/L (national drinking water standard for iAs in Bangladesh) [13]. In the USA and in Europe, the adverse health effects of chronic dietary exposure [9] to iAs are well documented. However, the USA and Europe have only recently recognized the need for regulatory limits on As due to a lack of data for the effects of As ingestion from food and due to the difficulty of assessing As exposure risk [9]. To fill this gap in the literature, we aimed to investigate the relationship between diet and total As exposure. 

Arsenic in cooked rice has been studied extensively. The As level in rice varies with the environmental conditions during cultivation [14,15,16] and with the method and water used for cooking [14,17,18,19]. Elevated As species in urine have been associated with high consumption of rice and rice-based products [20,21,22]. A survey of lifestyle and dietary habits in a Japanese population showed that higher than average consumption of rice (four or more bowls per day) was associated with a significantly higher average concentration of toenail As [23]. Particularly high As concentrations have been reported not only in cereals and cereal-based products [24,25,26,27,28], but also in fish and seafood [24,25,26,29,30] and vegetables [31,32,33]. While the majority of the As detected in cereal, grains, vegetables, and spices were iAs, fish and seafood contained mostly the organic forms of As and only a small portion of iAs [10].

Arsenic is a potential reproductive toxin since iAs and its methylated metabolites can readily cross the placenta [34,35]. In vivo studies show that even low levels of As exposure can induce oxidative stress [36], DNA methylation, altered gene expression, and free radical formation [37]. A systematic review of population-based studies found that high levels of groundwater iAs (≥50 µg/L) were associated with significantly increased risk of spontaneous abortion and stillbirth, and moderately increased risk of neonatal and infant mortality [38]. The most recent studies indicate that even low levels of iAs exposure in pregnant women are associated with lower than normal weight, length, chest circumference, and head circumference in newborns [38,39,40,41]. Thus, pregnant women are particularly vulnerable to adverse health effects of dietary As exposure [42]. However, the relationship between dietary patterns and As exposure has not been examined specifically in pregnant women. As part of our ongoing studies of the associations between As exposure and maternal and child health, this prospective study evaluated the association between long term diet and total As concentration in toenail clippings in a cohort of pregnant women in Bangladesh. 

## 2. Materials and Methods 

### 2.1. Study Population and Data Collection

This study utilized a prospective birth cohort from the administrative regions of Sirajdikhan and Pabna Sadar in Bangladesh. The purpose of studying this cohort was to investigate how chronic low dose As exposure affected reproductive outcomes during 2008–2011. A detailed description of the cohort analyzed in this study is available in the literature [43,44,45,46]. Briefly, the study recruited participants from areas known to have on average moderate but widely varying As concentrations in the ground water. Health care workers who had been trained by the Dhaka Community Hospital (DCH) Trust and who lived in villages served by DCH rural clinics assisted the researchers in identifying women eligible for the study and inviting them to participate. The eligibility criteria were age ≥ 18 years, ultrasound confirmed pregnancy at Gestation Week ≤ 16, use of tubewell-supplied groundwater as the primary drinking water source, intention to live at the current residence for the duration of pregnancy, intention to receive prenatal health care from DCH, and agreement to deliver either at DCH or at home with a DCH-trained midwife. Four visits were scheduled with each participant: time of enrollment (V1), Gestation Week 28 (V2), time of delivery (V3), and Postpartum Month 1 (V4). Data and sample collection in this study included sociodemographic characteristics (V1); lifestyle and personal habits (V1); medical records (V1, V2, and V4); drinking water history, e.g., drinking water source and water use habits, etc. (V1 and V2); dietary assessment (V2 and V4); toenail samples (V1 and V4); and water samples (V1 and V4). Participation in the study was incentivized by an offer of free prenatal care from DCH, including free multivitamin supplements. Each participant received in-home monthly checkups. At each checkup, multivitamin supplements were replenished and compliance with the prescribed multivitamin regimen was assessed. 

**Figure 1:**
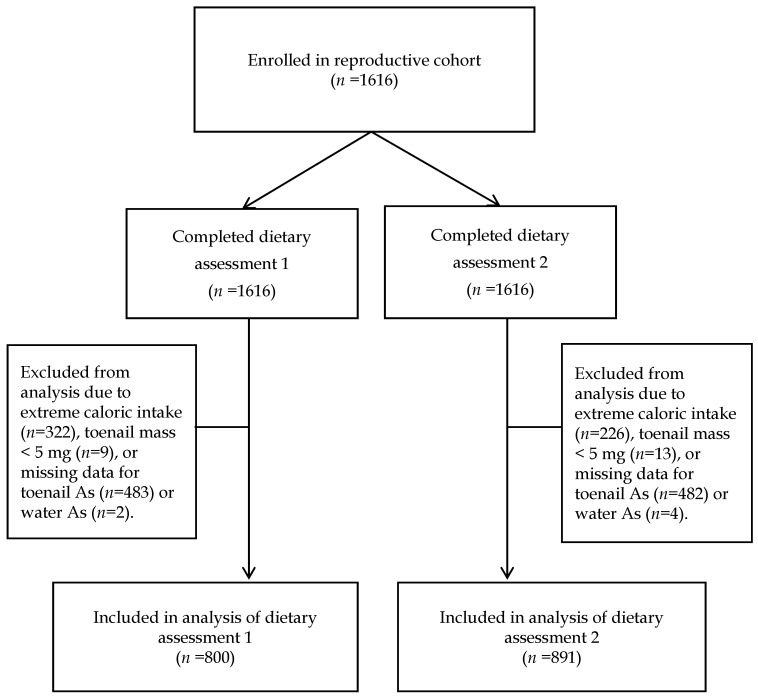
Study flow chart.

Figure 1 presents a flowchart of the participants (*n* = 1616) during the study. The cohort was enrolled between January 2008 and June 2011. Dietary assessment 1 was performed at V2, and dietary assessment 2 was performed at V4. In analysis of dietary assessment 1, the investigators excluded 816 participants due to missing data for toenail As (*n* = 483), missing data for water As (*n* = 2), extremely low or extremely high caloric intake defined as <500 kcal/day or >3500 kcal/day, respectively (*n* = 322), and toenail mass < 5 mg (*n* = 9). In analysis of dietary assessment 2, a total of 725 participants were excluded due to missing data for toenail As (*n* = 482), missing data for water As (*n* = 4), extremely low or extremely high caloric intake (*n* = 226), and toenail mass < 5 mg (*n* = 13). Thus, the final sample size was 800 and 891 for dietary assessment 1 and 2, respectively. Sensitivity analysis was performed using alternative exclusion criteria, i.e., total caloric intake below the 5th percentile and 95th percentile of the entire cohort. Informed consent was obtained from all study participants. The study protocol was approved by the institutional review boards of DCH and Harvard T.H. Chan School of Public Health (HSPH) (IRB number P11351, approved February 2008). 

### 2.2. Dietary Assessment

The FFQ used in this study was a locally-validated semi-quantitative written instrument covering the preceding 12-month period [47]. All participants (*n* = 1616), with the assistance of trained interviewers, completed the FFQ two times: at Gestation Week 28 (dietary assessment 1) and at Postpartum Month 1 (dietary assessment 2). The FFQ required participants to recall how frequently they had consumed 42 food items common in Bangladesh during the previous year. The food items were divided into five categories: (1) cereal and bread; (2) vegetables; (3) legumes, pulses, and seeds; (4) fish, poultry, meat, and eggs; and (5) milk based food items. Frequency of consumption was indicated on a 5-point scale ranging from “never” to “daily”. Participants were also shown photographs of locally-used plates, bowls, and serving utensils and then asked to indicate their typical portion sizes. Consumption of dietary supplements (e.g., vitamins) was also surveyed. As all participants received multivitamin supplements at V1, multivitamin intake was not analyzed for associations with toenail As concentration. All responses were converted to servings per day and grams per day using the midpoint of each frequency interval and assuming a 30-day month. Foods that were left blank were coded as not consumed [48]. Imputation procedures used in this study were performed as described in the literature [47]. Total energy intake was estimated using the most recent Food Composition Table for Bangladesh [49].

### 2.3. Arsenic Exposure Assessment

Arsenic has a high affinity for sulfhydryl groups and accumulates in keratin-rich tissues such as toenail [50]. Speciation analysis using HPLC-ICP-MS of aqueous toenail extract from other studies showed that As^III^, As^V^, and organic dimethylarsinate (DMA^V^) account for approximately 83%, 13%, and 8.5% of total toenail As concentration, respectively [51]. This study used toenail As as a biomarker of long-term As exposure in the past 2–18 months [32]. Each participant provided a sample of toenail clippings which were analyzed for total As concentrations using previously established protocols [44,45,46]. Briefly, clippings were collected using stainless steel scissors at V1 and V4, stored in labeled paper envelopes at room temperature, and shipped to HSPH for analysis. The toenail samples were processed by microwave-assisted acid digestion as described in Chen et al. [52]. Total As in the digested samples was analyzed by inductively coupled plasma (ICP) mass spectrometry (MS; Perkin Elmer, Shelton, CT, USA). A method bank and a certified reference material (CRM, human hair, Shanghai Institute of Nuclear Research, Academia Sinica, Shanghai, China) were included with each batch of samples used for digestion and analysis. Samples from the same study participant were digested and analyzed in the same batch. All analytical values were blank-corrected. To account for inter-batch differences in instrument performance, analytical values were multiplied by a factor equal to the inverse of the batch-specific percentage recovery in the CRM (mean percentage recovery for As was 76%). The mean limit of detection (LOD) for toenail As was 0.04 μg/g, and the relative standard deviation was 6.1%. Statistical comparisons of MS results for the toenail As samples and the CRM samples were performed as described in the literature. 

For each participant, samples of the primary source of drinking water were collected in 50 mL polypropylene tubes (BD Falcon, BD Bioscience, Bedford, MA, USA) at V1 and V4 and preserved in reagent grade nitric acid (Merck, Darmstadt, Germany) a pH < 2. The HSPH Environmental Laboratory Services (North Syracuse, New York, NY, USA) used USA EPA method 200.8 to perform ICP-MS of As in all water samples. Quality control testing of the instrument with spiked laboratory control sample (ICP, Analytical Mixture 12 Solution A, High Purity Standard, Charleston, SC, USA) yielded recoveries of 98% to 107%. For water samples that were below the LODs (*n* = 326 and *n* = 246 for V1 and V4, respectively), half the value of the LOD was used for statistical analysis.

### 2.4. Statistical Analysis 

Data were analyzed using SAS (version 9.3; SAS Institute Inc., Cary, NC, USA). Descriptive statistics were computed for all variables. Our previous study suggested that ingested water was the dominant source of As exposure in areas where the drinking water concentration exceeded the Bangladesh standard of 50 μg/L [13]. To examine whether water As level affected the association between water-corrected toenail As concentration and the intake level of each consumed food, we used a similar approach as described by Cottingham et al. [32]. Briefly, we included an interaction term between the intake rate of individual food item and an indicator variable for water As concentration (≤50 μg/L vs. >50 μg/L). For all foods, the interaction was statistically significant (α = 0.05), therefore, we grouped participants into high (>50 μg/L) and low exposure (≤50 μg/L) based on the As concentration in their primary source of drinking water. The *t* test, Fisher exact test, or analysis of variance was then used to compare the mean values of all variables between the two groups.

A generalized linear model was used to evaluate the relationship between toenail As concentrations and consumption of individual foods reported in the FFQ. Distributions of water As concentration, toenail As concentration, and individual food intake were skewed. Therefore, these variables were transformed according to their natural log (ln) form before regression modeling. “Crude” associations were considered to be associations between the consumption level of each food item and ln-transformed toenail As concentration after adjusting only for ln-transformed water As concentration within each strata of drinking water As concentration (≤50 μg/L vs. >50 μg/L). “Adjusted” associations accounted for additional covariates, including age, daily water and energy intake (estimated by FFQ), gender, betel nut chewing, tobacco chewing, smoking status, passive exposure to cigarette smoke, body mass index (BMI), and education level. Consumption measured by dietary assessment 1 at V2 was matched with toenail As and water As at V1; consumption measured by dietary assessment 2 at V4 were matched with toenail As and water As collected at V4. Reported effect estimates ($\hat{\beta }$) have units of ln((toenail As concentrations, μg/g)·(g/day)^−1^). For food items that were significantly associated with toenail As concentration, we tested the robustness of these associations by re-running models after and deleting apparent outliers; all associations remained significant.

To provide a more useful interpretation of parameter estimates, we employed a method previously reported in which we calculated the percentage change in toenail As concentration between the 5th and 95th percentiles of consumption for each consumed food [32]. Specifically, the percentage change in toenail As concentration was estimated using a back-transformation based on those participants who were nonsmokers, had a normal BMI (≥18.5 to <25 kg/m^3^), had attained a secondary education, and had a median value for water As concentration, age, water intake, and daily energy intake within their exposure group.

The false discovery rate (FDR) approach was used to account for type I error in conducting multiple statistical tests for each consumed food [53]. The *Q*-value (minimum FDR at which a test result may be considered statistically significant) was calculated from the combined list of *p*-values for the association with each food using the *Q*-value estimation package (Bioconductor version 3.4) in R [54]. A *Q*-value < 0.05 after correcting for multiple testing was considered statistically significant. 

For a clear visual depiction of multiple associations, the *corrplot* package in R was used to summarize all slope coefficients of the crude and adjusted models on a feature-expression heat-map [55]. 

## 3. Results

Among the 800 women in the cohort that completed dietary assessment 1 at V2, the median toenail As concentration was 1.6 μg/g, and the water As concentration ranged from 0.9 μg/L (lower quartile) to 35.3 μg/L (upper quartile) at V1 (Table 1). The 891 women who completed dietary assessment 2 at V4 had a median toenail As concentration of 1.2 μg/g, and water As concentration ranged from 1.3 μg/L (lower quartile) to 48.0 μg/L (upper quartile) at V4. The analysis included all food items in the dish-based FFQ except for three food items that no participant reported consuming (Tables S1 and S2). In the sensitivity analysis excluding participants with a daily caloric intake below the 5th percentile or above the 95th percentile of the entire cohort, fewer subjects were excluded from analyses (*n* = 162 and *n* = 113 for dietary assessment 1 and 2, respectively), however conclusions were not affected.

### 3.1. Comparison between Low and High Water As Exposure 

As expected, mean toenail As concentration was significantly lower in women with low (≤50 μg/L) water As exposure than in women with high (>50 μg/L) water As exposure. For both energy intake and water intake, average daily values were similar at dietary assessment 1 and dietary assessment 2. However, women with high water As had higher daily caloric intake and lower daily water intake compared to women with low water As (Table 1). Women with low water As and women with high water As were otherwise similar in age, betel nut use, tobacco chewing, cigarette smoking, BMI, and education level. 

### 3.2. Crude versus Adjusted Model

**Figure 2:**
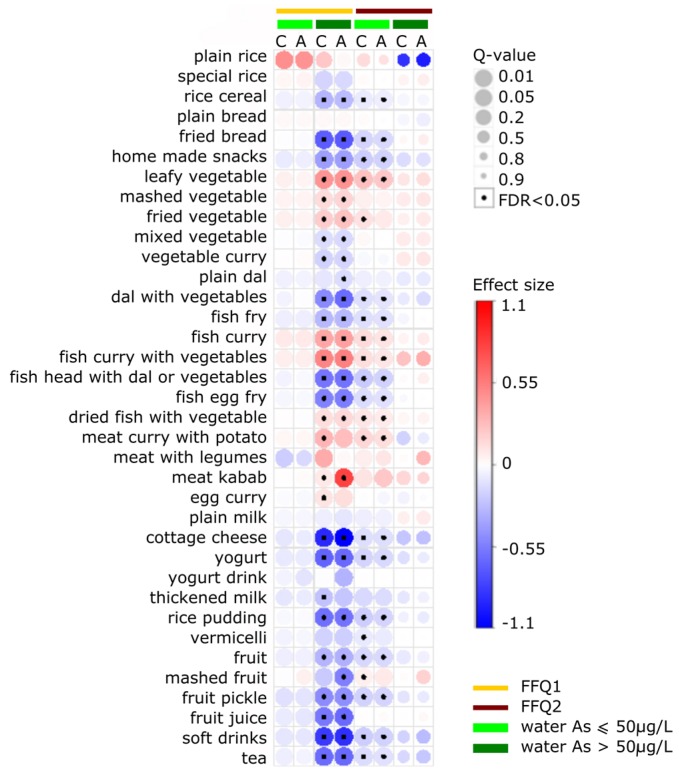
Associations between natural log-transformed food intake (g/day) and natural log-transformed toenail As concentration (μg/g) measured by the first and the second dietary assessment (FFQ1 and FFQ2, respectively) among women exposing to drinking water As concentration of >50 μg/L and ≤50 μg/L in Bangladesh. Effect size is the slope coefficients ($\hat{\beta }$) for each dietary item and has the unit of ln((toenail As concentrations, μg/g)·(g/day)^−1^). “C” stands for crude model adjusted for water As concentration only; “A” stands for adjusted model adjusted for water As level, sex, smoking in the living environment, chewing betel nut, BMI, daily water intake, daily energy intake, and education level. *Q*-value accounting for multiple comparisons using the false discovery rate (FDR = 0.05) method. (Quantitative values presented in the Supplementary Materials, Tables S1 and S2).

The crude and adjusted generalized linear models yielded similar results for most food items (Figure 2, Tables S1 and S2). The directions and strengths of associations were robust to the inclusion of potential confounding variables for individuals with low water As exposure. In individuals with high water As exposure, the directions of associations remained unchanged in comparisons of crude and adjusted models. However, as shown in Figure 2, for several food items (i.e., plain rice, meat kebab, and meat with legumes), the strength of the association changed after adjusting for potential confounders.

### 3.3. Comparison of Dietary Assessments 1 and 2

Mean toenail As concentration measured at V4 (at dietary assessment 2) was significantly lower than that at V1 (dietary assessment 1) (*p* = 0.05; Table 2). However, water As concentration and average daily caloric intake did not differ significantly between these two time points (*p* = 0.443 and *p* = 0.919, respectively). In dietary assessments 1 and 2, toenail As level showed a similar pattern of associations with average daily intake in all food items except for “plain rice” and “mashed fruit”. In subjects with high water As exposure (>50 μg/L), plain rice was positively associated with toenail As in dietary assessment 1 but was negatively associated with toenail As in dietary assessment 2 (Figure 2, Tables S1 and S2). However, no associations were statistically significant after adjustment for multiple testing. In subjects with high water As exposure, mashed fruit also had a significant positive association with toenail As in dietary assessment 1 but had a weak negative association with toenail As in dietary assessment 2. In individuals with low water As exposure (≤50 μg/L), the association between toenail As and consumption of mashed fruit was stronger in dietary assessment 2 than in dietary assessment 1.

### 3.4. Food Items Positively Associated with ln-Transformed Toenail As

Figure 2 shows that ln-transformed toenail As concentration had significant positive associations with ln-transformed consumption of plain rice, leafy vegetables, mashed vegetables, fried vegetables, fish curry, fish curry with vegetables, dried fish with vegetables, meat with potatoes, and meat kebab (Tables S1 and S2). Figure 2 further shows that, after correction for multiple testing, the positive association remained statistically significant for all food items except plain rice in individuals who had high water As exposure in dietary assessment 1 and in individuals with low water As exposure in dietary assessment 2. In the high water As exposure group, the food item that had the strongest positive association with toenail As was meat kebab (adjusted model $\hat{\beta }$ = 0.771 ± 0.122, *p* < 0.001 in dietary assessment 1). Table 2 shows that, according to this model, an increase in meat kebab consumption from 9 g/day (5th percentile) to 900 g/day (95th percentile) would incur a 33-fold increase in ln-transformed toenail As. In individuals with low water As exposure, the food items that had the strongest associations with toenail As were plain rice (adjusted model $\hat{\beta }$ = 0.445 ± 0.213, *p* = 0.037 in dietary assessment 1) and leafy vegetables (adjusted model $\hat{\beta }$ = 0.224 ± 0.057, *p* < 0.001 in dietary assessment 2), although the positive association for plain rice was not significant after adjusting for multiple comparison (*Q*-value > 0.05). The percent change in ln-transformed toenail As comparing the 5th to 95th percentile of consumption was predicted to be 13.65% and 36.45% for plain rice and leafy vegetable, respectively (Table 2).

### 3.5. Food Items Negatively Associated with ln-Transformed Toenail As

Many food items were negatively associated with toenail As. After correction for multiple comparisons, grains and bread based foods that had significant negative associations with toenail As included rice cereal, fried bread, and homemade snacks. In individuals with high water As exposure, an increase in consumption of rice cereal from 10 g/day (5th percentile) to 75 g/day (95th percentile) predicted a 62% decrease in toenail As. In the categories of legumes and pulses, milk based foods, fruits, and beverages, all food items showed negative associations with toenail As. In individuals with high water As exposure, the food items that had the strongest negative associations with toenail As were cottage cheese (adjusted model $\hat{\beta }$ = −1.053 ± 0.150, *p* < 0.001 in dietary assessment 1), soft drinks (adjusted model $\hat{\beta }$ = −0.861 ± 0.149, *p* < 0.001 in dietary assessment 1), and fried bread (adjusted model $\hat{\beta }$ = −0.679 ± 0.167, *p* < 0.001 in dietary assessment 1).

## 4. Discussion

This study used two successive dietary assessments (one at 28 weeks of gestation and the other at one month postpartum) to examine the relationship between long-term dietary patterns and As exposure in pregnant women. Total toenail As concentrations reflect the equilibrium of As distribution between the blood and nail during nail formation and extrusion [56] and provide a recapitulation of As exposure during the 2–18 months before sampling [32,57]. The validated dish-based FFQ used in this study captured dietary patterns in the previous 12 months; Thus, the exposure window captured by the FFQ in this study was matched with the As exposure window measured by the toenail clippings. Associations between food items and toenail As showed similar directions across the two time points. In dietary assessment 1, the associations were strongest among individuals with high water As exposure. In dietary assessment 2, the associations were strongest among individuals with low water As exposure. This difference suggests that accumulation of As through diet might differ before and during pregnancy. The ability of the body to methylate and excrete As is likely to have a positive association with the amount of As available for accumulation in the toenail. Notably, the crude and energy-adjusted regression results were similar, which suggests that, in terms As accumulation in the body, the total quantity of As consumed is more important than the quantity of As consumed in proportion to energy intake or body size. A previous study of the association between diet and toenail As in the US reported similar results [32].

Increased metabolism of As is among the many changes in metabolic physiology that occur during pregnancy to prepare the body for fetal growth. Data for metabolites of urinary As indicate that As methylation efficiency increases during pregnancy. Specifically, the shift from higher concentrations of the toxic As metabolite urinary monomethylarsonous acid (MMA) to higher concentrations of dimethylarsinous acid (DMA) after As exposure is more pronounced in pregnant women than in the general population [58,59,60,61,62]. Increased As methylation efficiency facilitates excretion of As from the body, which may explain the weaker association found in this study between food items and toenail As concentrations observed at dietary assessment 2 (which indicated dietary habits during pregnancy) compared to dietary assessment 1 (dietary habits prior to pregnancy and at early pregnancy). Another possible explanation for these weaker associations during pregnancy is related to increased folate consumption through the multivitamin supplement given to all participants after enrollment in this study. However, the effect of the supplement was probably small since increased methylation efficiency during pregnancy occurs independently of folate status [60]. Consumption of folate-related nutrients also affects As metabolism in the body. Studies in Bangladesh have reported that high intake of folate, cysteine, methionine, and calcium is associated with reduced urinary iAs [63] and with reduced incidence of As-related skin lesions [64]. During pregnancy, the low water As exposure group showed stronger associations between consumption of various food items and toenail As compared to the high water As exposure group. This suggests that changes in methylation efficiency and mechanisms of As removal from the body may differ between high and low As exposure. Further studies are needed to clarify how different levels of As exposure affect methylation efficiency during pregnancy. 

The population analyzed in this study represented a wide range of water As exposure levels. A previous study in Bangladesh suggested that food is a large contributor to total As exposure in areas with high As levels in the ground water (>50 μg/L) [13]. The current study similarly observed this interaction between water As exposure and diet. Thus, the study population was stratified into two groups based on water As exposure level. Compared to individuals with low water As exposure, those with high water As exposure tended to consume more rice, vegetables, fish, eggs, and meat but less rice cereal, homemade bread, and milk based food items. The difference in consumption levels did not explain the difference in the strength of associations between the two groups since the generalized linear model accounted for differences in consumption levels. However, the difference in strength of associations does suggest that As exposure affects As metabolism in a nonlinear fashion.

Arsenic concentration in food is dependent on aspects of the environment in which crops are cultivated, including location and season. For example, rice cultivated in As-contaminated soil and water showed higher level of As concentration, and rice crops irrigated with ground water showed significantly higher concentrations than rice cultivated at monsoon season [4,5]. The current study evaluated the long-term diet habit over the past 12 months, thus the effect of seasonal changes cannot be evaluated. Stratification of the analytical results by geographic area revealed no significant associations, possibly due to the lack of statistical power.

Elevated As levels in rice have been documented in several countries, including Bangladesh [4,5,15,65,66], the United States [67,68,69], and other countries in Asia [33,70]. Inorganic As contents in rice increased significantly after cooking. For example, a simulated gastrointestinal digestion study showed that iAs in cooked rice has 63%–99% bioavailability [71]. A longitudinal study of 18,470 adults in Bangladesh revealed a positive association between steamed rice consumption and creatinine-adjusted urinary total As, and similar findings were reported for a USA population and a Bangladeshi community living in the United Kingdom [21,25,72,73]. An FFQ survey performed in the USA further revealed a significant correlation between rice consumption and toenail As.

When the analyses in the current study focused only on the data for dietary assessment 1 (i.e., when data during pregnancy were excluded), rice consumption had a positive, albeit not statistically significant, association with toenail As. The association was stronger in participants exposed to low water As levels. In this group, an increase in rice consumption from the 5th to the 95th percentile predicted a 13.65% increase in toenail As concentration in nonsmokers who had a normal BMI, secondary education level, mean age, mean daily water and energy intake, and median water As concentration. Notably, toenail As was negatively associated with consumption of other grains, cereals and cereal products such as rice cereal, fried bread and homemade snacks. Possible explanations for this negative association include the low water content and low As level in these food items as well as the lower consumption of rice relative to consumption of grains, cereal, and bread. There is a need for further study to determine whether these food items are effective and feasible alternatives to plain rice in areas known to have As-contaminated water.

Vegetables, fish and meat items also revealed positive associations with toenail As. Laboratory analysis of vegetables commonly included in the Bangladeshi diet, such as *kachu sak* (*Colocasia antiquorum*), potatoes (*Solanum tuberisum*), and *kalmi sak* (*Ipomoea reptoms*), showed elevated As levels in these food items. However, the elevation of As varied by cultivar and area of cultivation [31]. Another survey performed in Shanxi, China, found that 77% of ground water samples exceeded the WHO guideline of 10 μg/L As. Several vegetables revealed As levels exceeding 1 μg/g, including cucumbers, tomatoes, eggplant, scallions, beans, and cabbage [33]. In a New Hampshire population known to have As-contaminated drinking water, Brussels sprouts consumption was positively associated with toenail As level [32]. The total As concentration in vegetables, predominately iAs, varies by the plant tissue type [74]. Generally, the order of As accumulation in plant tissues is, from first to last, root, stem, leaf, and grain. In vivo feeding studies show that the As bioavailability of edible plant tissues ranges from 45% (chard) to 95% (mung bean) [74]. Available data for the association between vegetable consumption and As exposure are limited. A Pakistan study reported that iAs exposure from vegetable consumption is not a major cancer risk factor [75]. While our data showed that some vegetable food items (leafy vegetables, mashed vegetables, and fried vegetables) are positively associated with toenail As, the association is likely attributable to the high water As content of the vegetable items or their preparation method. Further studies are needed for a more comprehensive understanding of the association between vegetable consumption and As uptake. 

The positive association between fish consumption and toenail As is poorly understood. In the USA, fish and seafood consistently show higher total As concentrations compared to other food items and are the main contributors to As intake [26,29,76,77]. Recent studies show that consumption of fish is positively associated with As biomarkers in both urine [77,78] and toenail tissue [56]. A possible explanation for this association is that, although iAs contributes a relatively small percentage of total As in seafood (approximately 1.5% and 20% in fish and shellfish, respectively), the iAs in seafood may still contribute appreciably to total As exposure [25]. New evidence also suggests that reactive trivalent arsenic intermediates, which have high affinity for the cysteine component in nails, may be formed during the bioconversion of arsenosugars and arsenolipids in seafood to urinary excretion of DMA [79]. However, more research is needed to clarify the possible mechanism behind this.

Consumptions of fruits and most milk based food items were negatively associated with toenail As. Fruits are important sources of micronutrients, fiber, and antioxidants (e.g., vitamins E and C), which protect the body from oxidative stress induced by As exposure [80]. Therefore, consumption of more fruits may result in lower toenail As due to better overall nutrition. Another explanation for these negative associations may be related to factors which mitigate As absorption in the gastrointestinal tract [25], and which could be the case for milk based food items that contain high levels of fat and protein. A study in a New Hampshire population with low groundwater As was the first to report that dietary lipids are negatively associated with toenail As [81]. In another study, an in vitro simulation of the human gastrointestinal microbial system showed that a typically high fat, high protein western diet results in lower As bioaccessibility compared with a typical high fiber, low fat Asian diet (63.4% versus 81.2%, respectively) [82]. Animal models show that interactions between As and invertebrate and vertebrate lipid particles cause As detoxification via cell-free or sequestration mechanisms [83]. Additional studies are needed to clarify the roles of lipid consumption in As detoxification and As accumulation in the human body. 

One strength of this study is the use of a validated FFQ [47]. In comparison with the six-day food diary conducted a previous study [47], the FFQ showed fair to strong correlations for grain (Spearman correlation = 0.35); vegetables (Spearman correlation = 0.25); legumes, pulses, and seeds (Spearman correlation = 0.34); fish, poultry, meat, and eggs (Spearman correlation = 0.42); milk (Spearman correlation = 0.66); and fruits (Spearman correlation = 0.79). Another strength is the use of a prospective cohort design, which revealed the temporal sequence of exposure and outcome and avoided selection bias at enrollment. This study evaluated heavy metals exposure not only by performing a comprehensive dietary assessment, but also by collecting water samples during each household visit; by administering comprehensive questionnaires on lifestyle, family medical history, and water use; and by collecting biospecimens. Third, a rigorously-developed gold standard ICP-MS method was used to detect toenail As concentration. Comprehensive comparisons with other As biomarkers show that toenail As has good sensitivity and specificity [12,45]. Finally, analysis of a population representing a wide range of As exposure levels provided adequate power to observe associations with As exposure.

In large epidemiologic studies, the FFQ is considered the most feasible way to capture long-term dietary habits [84]. However, using a FFQ to estimate consumption is subject to important sources of error. The average daily energy intake in the current study was higher than that reported by the Bangladesh Household Income and Expenditure Survey (HIES) [85]. Preferred food items or food items with known health benefits are likely to be over-reported in an FFQ survey [47,86]. In the current study, the higher average daily energy intake obtained by the FFQ in comparison with the HIES probably resulted from high consumption of plain rice, which is the main source of caloric intake in rural Bangladesh. Additionally, the subjects of this study may have tended to over-report consumption of rice because it symbolizes better economic status. Nevertheless, considering that most participants had similar socioeconomic status (most had a secondary education or lower), over-reporting was expected to be non-differential and was therefore expected to bias the association toward the null. This over-reporting bias may also explain the small variation in plain rice consumption observed in our study population, which may in turn contribute to the non-significant association observed between rice consumption and toenail As concentration.

Another limitation of the FFQ is its relative lack of precision in comparison with other dietary assessment methods. Potentially more precise instruments, such as the diet record or 24-h recall, are available but were not feasible in this study due to their laboriousness, cost, and unsuitability for measuring long-term intake. While we matched the exposure window captured by the FFQ with the As exposure window measured by the toenail clippings, use of the FFQ may still incur recall bias, such that one’s recent diet may influence reporting of consumption levels. In comparison with precision of the toenail As level, the precision of dietary measurements was lower, violating the convention that predictor variables should be more precise than the response variable in regression modeling [32]. Fortunately, this violation tends to bias results toward the null, so our findings should still be valid [87].

## 5. Conclusions

In this study, toenail As concentration, which is a well-recognized biomarker of As exposure, showed positive associations with several food items commonly consumed in Bangladesh, including leafy vegetables (*sak*), mashed vegetables (*bhorta*), fried vegetables (*bhaji*), fish curry (*mac her jhole*), fish curry with vegetables, dried fish with vegetables, meat with potatoes, and meat kebab. In contrast, consumption of rice cereal (*chira*, *muri*, *khoi*, and *murki*), fruits, and many milk based food items were negatively associated with toenail As, possibly due to disrupted As absorption in the gastrointestinal tract or due to reduced As intake. Another possible explanation is that these negative associations indicate a specific dietary pattern (i.e., one marked by low consumption of plain rice). The directions of association were similar in groups with exposure to high As in drinking water and groups with exposure to low As in drinking water, however, the strengths of associations markedly differed between these groups. Comparisons of the strength of association at different stages of pregnancy suggests a change in the mechanisms of As detoxification and excretion from the body during pregnancy. Further studies are needed to explore mechanisms behind the effects of diet on As absorption and excretion and on As metabolism during pregnancy.

## Figures and Tables

**Table 1:** Demographic characteristics of the study population across the two dietary assessment periods.

Group	1st Dietary Assessment (FFQ1)							2nd Dietary Assessment (FFQ2)						
	Total (*n* = 800)		Water [As] ≤ 50 μg/L (*n* = 629)		Water [As] > 50 μg/L (*n* = 171)			Total (*n* = 891)		Water [As] ≤ 50 μg/L (*n* = 683)		Water [As] > 50 μg/L (*n* = 208)		
Continuous Variables	Mean	SD	Mean	SD	Mean	SD	*p*-Value *	Mean	SD	Mean	SD	Mean	SD	*p*-Value *
Age (years)	23.0	4.3	23.0	4.3	23.2	4.3	0.590	23.0	4.2	23.0	4.3	23.1	3.9	0.764
Toenail [As] (μg/g)	3.3	4.6	2.3	3.5	7.1	5.8	<0.001	2.7	4.1	1.8	3.3	5.8	4.7	<0.001
Water [As] (μg/L)	45.8	111.6	6.8	11.1	188.9	178.6	<0.001	50.0	113.2	8.6	12.7	210.6	193.1	<0.001
Water intake (L/day)	2.1	0.5	2.2	0.5	2.0	0.4	<0.001	2.2	0.4	2.2	0.4	2.1	0.4	0.002
Energy intake (kcal/day)	2804.2	417.5	2787.6	416.7	2865.0	416.1	0.032	2806.2	396.1	2777.5	389.9	2900.7	402.4	<0.001
**Dichotomous Variables**	***N***	**%**	***N***	**%**	***N***	**%**		***N***	**%**	***N***	**%**	***N***	**%**	
Sex (female)	800	100	629	100	171	100	1.000	891	100.0	683	100.0	208	100.0	1.000
Betel nut	6	0.8	4	0.6	2	1.2	0.614	8	0.9	6	0.9	2	1.0	0.591
Chew tobacco	4	0.5	2	0.3	2	1.2	0.202	6	0.7	4	0.6	2	1.0	0.427
Environmental smoke	339	42.4	251	39.9	88	51.5	0.010	362	40.6	257	37.6	105	50.5	0.001
Smoker	0	0.0	0	0.0	0	0.0	1.000	0	0.0	0	0.0	0	0.0	1.000
**Categorical Variables**	***N***	**%**	***N***	**%**	***N***	**%**		***N***	**%**	***N***	**%**	***N***	**%**	
BMI							0.209	-	-	-	-	-	-	0.016
Underweight (<18.5)	231	28.9	173	27.5	58	33.9		241	27.0	168	24.6	73	27.0	
Normal (18.5 ≤ BMI < 25)	497	62.1	395	62.8	102	59.6		569	63.9	447	65.4	122	63.9	
Overweight (25 ≤ BMI < 30)	65	8.1	56	8.9	9	5.3		71	8.0	59	8.6	12	8.0	
Obese (≥30)	7	0.9	5	0.8	2	1.2		10	1.1	9	1.3	1	1.1	
Education							0.378	-	-	-	-	-	-	0.499
Illiterate	12	1.5	10	1.6	2	1.2		10	1.1	8	1.2	2	1.0	
Able to write	106	13.3	81	12.9	25	14.6		125	14.0	94	13.7	31	14.9	
Primary education	271	33.9	218	34.7	53	31.0		292	32.8	236	34.6	56	26.9	
Secondary education	385	48.1	298	47.4	87	50.9		431	48.4	319	46.7	112	53.9	
Higher secondary education	24	3.0	21	3.3	3	1.8		27	3.0	21	3.1	6	2.9	
College/graduate	1	0.1	0	0.0	1	0.6		4	0.4	3	0.4	1	0.5	
Post-graduate	1	0.1	1	0.2	0	0.0		2	0.2	2	0.3	0	0.0	

* Comparing water [As] ≤ 50 μg/L vs. >50 μg/L using two sample *t* test for continuous variables, Fisher’s exact test for dichotomous variables and Chi-square test for categorical variables.

**Table 2:** Mean daily intake level for each food item and the percent change in toenail As concentration comparing the 5th to the 95th percentile of intake level.

Food Items	1st Dietary Assessment						2nd Dietary Assessment					
	Water [As] ≤ 50 μg/L (*n* = 629)			Water [As] > 50 μg/L (*n* = 171)			Water [As] ≤ 50 μg/L (*n* = 683)			Water [As] > 50 μg/L (*n* = 208)		
	Daily Intake ∞		% Change Toenail As ^⌘^	Daily Intake ∞		% Change Toenail As ^⌘^	Daily Intake ∞		% Change Toenail As ^⌘^	Daily Intake ∞		% Change Toenail As ^⌘^
	Mean	STD		Mean	STD		Mean	STD		Mean	STD	
Grain, Cereal, Bread												
Plain rice *(Bhaat, Panta bhaat)*	1355.14	160.79	13.65	1396.38	151.84	0.79	1331.07	150.00	4.95	1369.2	107.1	−3.22
Special rice *(Khichuri, Pulao, Biriyani)*	25.83	30.67	6.47	25.04	30.21	−26.23	25.51	27.68	−0.48	24.6	23.7	9.89
Rice cereal *(Chira, Muri, Khoi, Murki)*	68.60	56.93	−16.40	29.12	42.02	−62.00 *	62.42	52.53	−21.67 *	14.5	20.3	−8.43
Plain bread *(Atta ruti, Pau ruti)*	84.60	92.94	6.28	81.20	79.88	7.73	98.22	103.74	−1.84	73.5	65.7	−13.32
Fried bread *(Porota, Luchi)*	22.37	20.17	−3.46	12.67	6.24	−61.80 *	33.52	25.52	−27.46 *	14.5	12.3	8.76
Home made snacks *(Pitha-puli)*	31.96	32.74	−15.21	17.23	22.38	−61.77 *	32.06	25.40	−37.83 *	12.1	8.3	−21.34
Vegetable												
Leafy vegetable *(Sak)*	56.03	41.31	6.29	80.86	41.32	85.95 *	54.54	37.07	36.43 *	91.5	35.7	9.35
Mashed vegetable *(Bhorta)*	24.87	18.92	13.05	29.70	21.10	59.42 *	20.16	20.86	12.59	29.7	21.3	30.41
Fried vegetable *(Bhaji)*	50.82	32.08	15.33	66.77	34.89	97.20 *	49.04	31.13	27.93	71.4	36.8	28.30
Mixed vegetable *(Labra)*	41.88	34.47	−4.79	43.66	44.04	−39.72 *	35.00	33.09	−0.10	38.7	41.6	31.97
Vegetable Curry (Torkarir jhole)	51.91	36.11	3.13	51.96	51.61	−45.60 *	47.47	32.28	−4.94	39.3	44.7	38.48
Legumes, Pulses, Seeds												
Plain dal	67.82	38.63	−12.83	58.72	42.30	−39.28 *	64.22	30.46	−12.07	57.6	37.7	−25.19
Dal with vegetables	10.77	5.00	−0.11	9.99	3.94	−49.19 *	44.52	34.67	−28.83 *	7.8	9.8	−12.57
Fish, Poultry, Meat, Egg												
Fish Fry *(Mach bhaji)*	194.79	128.82	−18.61	101.51	101.42	−54.10 *	197.75	147.83	−30.76 *	56.3	65.7	−1.26
Fish curry *(Mach er jhole)*	126.17	101.19	36.93	211.48	92.26	116.90 *	119.67	98.76	47.27 *	217.8	83.6	17.76
Fish curry with vegetable	156.39	121.88	21.21	271.76	93.54	189.10 *	160.99	128.42	24.30 *	301.9	61.3	26.18
Fish head with dal or vegetables	12.64	10.83	−5.62	7.05	5.07	−74.44 *	10.98	11.12	−28.62 *	5.1	3.3	13.42
Fish egg fry (Maccher dim bhaji)	4.87	4.00	−6.83	2.00	2.44	−77.12 *	8.05	8.25	−35.29*	1.3	1.7	0.26
Dried fish with vegetable	94.79	116.21	−1.01	157.31	128.09	64.23 *	102.32	129.05	38.37 *	210.9	122.9	13.71
Meat curry with potato	32.60	34.24	9.51	36.34	19.05	20.64	24.65	20.37	41.15 *	37.3	25.6	−5.22
Meat with legumes *(Halim)*	12.28	4.06	−13.98	12.18	3.69	2.98	16.64	13.17	32.98	23.7	15.2	102.25
Meat kebab	171.36	555.09	9.20	200.26	415.79	3321.24 *	96.46	66.16	54.79	130.9	82.9	37.86
Egg curry *(Dim er jhole)*	98.57	84.90	−7.24	134.87	107.91	43.85	96.42	71.96	−8.45	139.9	103.1	−3.31
Milk												
Plain milk *(Doodh)*	127.10	92.50	−13.78	81.47	73.91	−32.26	132.31	87.45	−13.33	70.7	58.2	36.04
Cottage cheese *(chana)*	2.45	1.24	−11.58	1.60	0.94	−79.78 *	3.88	3.28	−32.93 *	0.9	1.0	−28.02
Yogurt *(Doi)*	5.20	4.50	−13.87	2.88	2.66	−71.94 *	4.74	3.17	−25.05 *	1.7	1.0	−9.96
Yogurt drink *(Ghole, Matha, Borhani)*	7.13	4.89	−21.63	5.97	3.71	−44.92	NA	NA	NA	NA	NA	NA
Thickened milk *(Khoa, kheer)*	11.83	7.83	−10.20	10.08	5.99	−27.50	11.40	4.86	−18.26	9.8	7.5	−15.66
Rice pudding *(Payesh)*	15.07	11.86	−2.96	8.54	5.48	−68.47 *	14.85	15.51	−32.05 *	6.8	7.2	−9.59
Vermicelli *(Semai)*	30.15	27.62	−9.81	22.91	21.91	−23.35	33.42	30.39	−18.49	20.9	15.4	1.16
Fruits												
Fruit	78.85	62.33	−17.87	32.39	39.69	−58.55 *	79.74	55.88	−38.10 *	37.3	109.8	−13.78
Mashed fruit *(Bhorta)*	2.32	1.12	7.93	2.53	4.20	−49.13 *	5.34	9.08	41.70	9.6	10.1	77.70
Fruit pickle *(Aachar)*	1.67	3.15	−24.05	1.26	1.79	−78.20 *	1.95	1.61	−37.58 *	0.7	0.6	−15.64
Beverages												
Fruit juice	26.09	19.30	−21.87	12.85	12.28	−76.25 *	4071.49	36,352.71	15.73	2343.3	18,866.8	21.76
Soft drinks	17.33	9.56	−15.11	10.22	5.78	−75.45 *	16.42	10.75	−28.95 *	7.0	2.3	−20.71
Tea	36.63	27.29	−9.65	21.58	23.80	−77.18 *	40.64	31.81	−26.73 *	11.7	10.0	−20.03

∞: Unit in ln(gram/day); ^⌘^: Comparing consumers of 5th percentile of intake with those of 95th percentile of intake for each individual food item. The comparison is done among nonsmoker, with normal BMI and secondary education level at the mean age, water intake, and daily energy intake and the median water As concentration of each exposure group using the beta coefficient estimate of adjusted model (Unit: percent change in g/day); *: *Q*-value < 0.05.

## Supplementary Materials

The following are available online at http://www.mdpi.com/2072-6643/9/4/420/s1, Table S1: The association of food intake with toenail arsenic concentration using linear regression for dietary assessment 1, Table S2: The association of food intake with toenail arsenic concentration using linear regression for dietary assessment 2.

## References

[1] WHO Exposure to Arsenic: A Major Public Health Concern. http://www.who.int/ipcs/assessment/public_health/arsenic/en/.

[2] Agency for Toxic Substances and Disease Registry (ATSDR) (2000). Agency for Toxic Substances and Disease Registry: Arsenic Toxicity: Environmental Alert.

[3] Tseng C.-H. (2009). A review on environmental factors regulating arsenic methylation in humans. Toxicol. Appl. Pharmacol..

[4] Meharg A.A., Rahman M.M. (2003). Arsenic contamination of Bangladesh paddy field soils: Implications for rice contribution to arsenic consumption. Environ. Sci. Technol..

[5] Duxbury J.M., Mayer A.B., Lauren J.G., Hassan N. (2003). Food chain aspects of arsenic contamination in Bangladesh: Effects on quality and productivity of rice. J. Environ. Sci. Health A Toxic Hazard. Subst. Environ. Eng..

[6] MacIntosh D.L., Spengler J.D., Ozkaynak H., Tsai L., Ryan P.B. (1996). Dietary exposures to selected metals and pesticides. Environ. Health Perspect..

[7] Pizarro I., Gómez M.M., Fodor P., Palacios M.A., Cámara C. (2004). Distribution and biotransformation of arsenic species in chicken cardiac and muscle tissues. Biol. Trace Elem. Res..

[8] Lasky T., Sun W., Kadry A., Hoffman M.K. (2004). Mean total arsenic concentrations in chicken 1989–2000 and estimated exposures for consumers of chicken. Environ. Health Perspect..

[9] Cubadda F., Jackson B.P., Cottingham K.L., Van Horne Y.O., Kurzius-Spencer M. (2017). Human exposure to dietary inorganic arsenic and other arsenic species: State of knowledge, gaps and uncertainties. Sci. Total Environ..

[10] Borak J., Hosgood H.D. (2007). Seafood arsenic: Implications for human risk assessment. Regul. Toxicol. Pharmacol..

[11] European Food Safety Authority (2009). Scientific Opinion on Arsenic in Food. EFSA J..

[12] Kile M.L., Houseman E.A., Breton C.V., Quamruzzaman Q., Rahman M., Mahiuddin G., Christiani D.C. (2007). Association between total ingested arsenic and toenail arsenic concentrations. J. Environ. Sci. Health A Toxic Hazard. Subst. Environ. Eng..

[13] Kile M.L., Houseman E.A., Breton C.V., Smith T., Quamruzzaman Q., Rahman M., Mahiuddin G., Christiani D.C. (2007). Dietary arsenic exposure in bangladesh. Environ. Health Perspect..

[14] Bae M., Watanabe C., Inaoka T., Sekiyama M., Sudo N., Bokul M.H., Ohtsuka R. (2002). Arsenic in cooked rice in Bangladesh. Lancet.

[15] Alam M.G.M., Snow E.T., Tanaka A. (2003). Arsenic and heavy metal contamination of vegetables grown in Samta village, Bangladesh. Sci. Total Environ..

[16] Roychowdhury T., Tokunaga H., Ando M. (2003). Survey of arsenic and other heavy metals in food composites and drinking water and estimation of dietary intake by the villagers from an arsenic-affected area of West Bengal, India. Sci. Total Environ..

[17] Signes A., Mitra K., Burló F., Carbonell-Barrachina A.A. (2008). Effect of cooking method and rice type on arsenic concentration in cooked rice and the estimation of arsenic dietary intake in a rural village in West Bengal, India. Food Addit. Contam. A.

[18] Gray P.J., Conklin S.D., Todorov T.I., Kasko S.M. (2016). Cooking rice in excess water reduces both arsenic and enriched vitamins in the cooked grain. Food Addit. Contam. A.

[19] Carey M., Jiujin X., Gomes Farias J., Meharg A.A., Meharg A., Williams P., Adamako E., Lawgali Y., Deacon C., Villada A. (2015). Rethinking Rice Preparation for Highly Efficient Removal of Inorganic Arsenic Using Percolating Cooking Water. PLoS ONE.

[20] Cascio C., Raab A., Jenkins R.O., Feldmann J., Meharg A.A., Haris P.I. (2011). The impact of a rice based diet on urinary arsenic. J. Environ. Monit..

[21] Gilbert-Diamond D., Cottingham K.L., Gruber J.F., Punshon T., Sayarath V., Gandolfi A.J., Baker E.R., Jackson B.P., Folt C.L., Karagas M.R. (2011). Rice consumption contributes to arsenic exposure in US women. Proc. Natl. Acad. Sci. USA.

[22] Wei Y., Zhu J., Nguyen A. (2014). Rice consumption and urinary concentrations of arsenic in US adults. Int. J. Environ. Health Res..

[23] Tabata H., Anwar M., Horai S., Ando T., Nakano A., Wakamiya J., Koriyama C., Nakagawa M., Yamada K., Akiba S. (2006). Toenail arsenic levels among residents in Amami-Oshima Island, Japan. Environ. Sci..

[24] Dabeka R.W., McKenzie A.D., Lacroix G.M., Cleroux C., Bowe S., Graham R.A., Conacher H.B., Verdier P. (1993). Survey of arsenic in total diet food composites and estimation of the dietary intake of arsenic by Canadian adults and children. J. AOAC Int..

[25] MacIntosh D.L., Williams P.L., Hunter D.J., Sampson L.A., Morris S.C., Willett W.C., Rimm E.B. (1997). Evaluation of a food frequency questionnaire-food composition approach for estimating dietary intake of inorganic arsenic and methylmercury. Cancer Epidemiol. Biomark. Prev..

[26] Schoof R.A., Yost L.J., Eickhoff J., Crecelius E.A., Cragin D.W., Meacher D.M., Menzel D.B. (1999). A market basket survey of inorganic arsenic in food. Food Chem. Toxicol..

[27] Meharg A.A., Deacon C., Campbell R.C.J., Carey A.-M., Williams P.N., Feldmann J., Raab A. (2008). Inorganic arsenic levels in rice milk exceed EU and US drinking water standards. J. Environ. Monit..

[28] Meharg A.A., Sun G., Williams P.N., Adomako E., Deacon C., Zhu Y.-G., Feldmann J., Raab A. (2008). Inorganic arsenic levels in baby rice are of concern. Environ. Pollut..

[29] Tao S.S.-H., Michael Bolger P. (1999). Dietary arsenic intakes in the United States: FDA Total Diet Study, September 1991–December 1996. Food Addit. Contam..

[30] DeCastro B.R., Caldwell K.L., Jones R.L., Blount B.C., Pan Y., Ward C., Mortensen M.E., Chen C., Hsueh Y., Lai M. (2014). Dietary Sources of Methylated Arsenic Species in Urine of the United States Population, NHANES 2003–2010. PLoS ONE.

[31] Das H.K., Mitra A.K., Sengupta P.K., Hossain A., Islam F., Rabbani G.H. (2004). Arsenic concentrations in rice, vegetables, and fish in Bangladesh: A preliminary study. Environ. Int..

[32] Cottingham K.L., Karimi R., Gruber J.F., Zens M.S., Sayarath V., Folt C.L., Punshon T., Morris J.S., Karagas M.R. (2013). Diet and toenail arsenic concentrations in a New Hampshire population with arsenic-containing water. Nutr. J..

[33] Cui J., Shi J., Jiang G., Jing C. (2013). Arsenic Levels and Speciation from Ingestion Exposures to Biomarkers in Shanxi, China: Implications for Human Health. Environ. Sci. Technol..

[34] Hood R.D., Vedel-Macrander G.C., Zaworotko M.J., Tatum F.M., Meeks R.G. (1987). Distribution, metabolism, and fetal uptake of pentavalent arsenic in pregnant mice following oral or intraperitoneal administration. Teratology.

[35] Hood R.D., Vedel G.C., Zaworotko M.J., Tatum F.M., Meeks R.G. (1988). Uptake, distribution, and metabolism of trivalent arsenic in the pregnant mouse. J. Toxicol. Environ. Health.

[36] Flora S.J.S. (2011). Arsenic-induced oxidative stress and its reversibility. Free Radic. Biol. Med..

[37] Wlodarczyk B.J., Bennett G.D., Calvin J.A., Finnell R.H. (1996). Arsenic-induced neural tube defects in mice: Alterations in cell cycle gene expression. Reprod. Toxicol..

[38] Quansah R., Armah F.A., Essumang D.K., Luginaah I., Clarke E., Marfo K., Cobbina S.J., Nketiah-Amponsah E., Namujju P.B., Obiri S. (2015). Association of Arsenic with Adverse Pregnancy Outcomes-Infant Mortality: A Systematic Review and Meta-Analysis. Environ. Health Perspect..

[39] Guan H., Piao F., Zhang X., Li X., Li Q., Xu L., Kitamura F., Yokoyama K. (2012). Prenatal exposure to arsenic and its effects on fetal development in the general population of Dalian. Biol. Trace Elem. Res..

[40] Xu L., Yokoyama K., Tian Y., Piao F.-Y., Kitamura F., Kida H., Wang P. (2011). Decrease in birth weight and gestational age by arsenic among the newborn in Shanghai, China. Nihon Koshu Eisei Zasshi.

[41] Smith A.H., Hopenhayn-Rich C., Bates M.N., Goeden H.M., Hertz-Picciotto I., Duggan H.M., Wood R., Kosnett M.J., Smith M.T. (1992). Cancer risks from arsenic in drinking water. Environ. Health Perspect..

[42] Kordas K., Lönnerdal B., Stoltzfus R.J. (2007). Interactions between nutrition and environmental exposures: Effects on health outcomes in women and children. J. Nutr..

[43] Huyck K.L., Kile M.L., Mahiuddin G., Quamruzzaman Q., Rahman M., Breton C.V., Dobson C.B., Frelich J., Hoffman E., Yousuf J. (2007). Maternal Arsenic Exposure Associated With Low Birth Weight in Bangladesh. J. Occup. Environ. Med..

[44] Kile M.L., Rodrigues E.G., Mazumdar M., Dobson C.B., Diao N., Golam M., Quamruzzaman Q., Rahman M., Christiani D.C. (2014). A prospective cohort study of the association between drinking water arsenic exposure and self-reported maternal health symptoms during pregnancy in Bangladesh. Environ. Health.

[45] Rodrigues E.G., Kile M., Dobson C., Amarasiriwardena C., Quamruzzaman Q., Rahman M., Golam M., Christiani D.C. (2015). Maternal–infant biomarkers of prenatal exposure to arsenic and manganese. J. Expo. Sci. Environ. Epidemiol..

[46] Kile M.L., Cardenas A., Rodrigues E., Mazumdar M., Dobson C., Golam M., Quamruzzaman Q., Rahman M., Christiani D.C. (2016). Estimating Effects of Arsenic Exposure During Pregnancy on Perinatal Outcomes in a Bangladeshi Cohort. Epidemiology.

[47] Lin P.-I., Bromage S., Mostofa M., Allen J., Oken E., Kile M., Christiani D. (2017). Validation of a Dish-Based Semiquantitative Food Questionnaire in Rural Bangladesh. Nutrients.

[48] Michels K.B., Willett W.C. (2009). Self-administered semiquantitative food frequency questionnaires: Patterns, predictors, and interpretation of omitted items. Epidemiology.

[49] Shaheen N., Rahim A.T., Banu M.C.P., Bari L., Tukun B., Mannan M., Bhattacharjee L., Stadlmayr B. (2013). Food Composition Table for Bangladesh.

[50] Karagas M.R., Morris J.S., Weiss J.E., Spate V., Baskett C., Greenberg E.R. (1996). Toenail samples as an indicator of drinking water arsenic exposure. Cancer Epidemiol. Biomarkers Prev..

[51] Button M., Jenkin G.R.T., Harrington C.F., Watts M.J. (2009). Human toenails as a biomarker of exposure to elevated environmental arsenic. J. Environ. Monit..

[52] Chen K.L., Amarasiriwardena C.J., Christiani D.C. (1999). Determination of total arsenic concentrations in nails by inductively coupled plasma mass spectrometry. Biol. Trace Elem. Res..

[53] Catelan D., Biggeri A. (2010). Multiple testing in disease mapping and descriptive epidemiology. Geospat. Health.

[54] Bass J., Dabney A., Robinson D. (2015). Qvalue: *Q*-Value Estimation for False Discovery Rate Control. R Packag. http://github.com/jdstorey/qvalue/.

[55] Haarman B.C.M., Riemersma-Van der Lek R.F., Nolen W.A., Mendes R., Drexhage H.A., Burger H. (2015). Feature-expression heat maps—A new visual method to explore complex associations between two variable sets. J. Biomed. Inform..

[56] Rees J.R., Sturup S., Chen C., Folt C., Karagas M.R. (2007). Toenail mercury and dietary fish consumption. J. Expo. Sci. Environ. Epidemiol..

[57] Chen K.-L.B., Amarasiriwardena C.J., Christiani D.C. (1999). Determination of total arsenic concentrations in nails by inductively coupled plasma mass spectrometry. Biol. Trace Elem. Res..

[58] Hopenhayn C., Huang B., Christian J., Peralta C., Ferreccio C., Atallah R., Kalman D. (2003). Profile of urinary arsenic metabolites during pregnancy. Environ. Health Perspect..

[59] Lindberg A.-L., Ekström E.-C., Nermell B., Rahman M., Lönnerdal B., Persson L.-Å., Vahter M. (2008). Gender and age differences in the metabolism of inorganic arsenic in a highly exposed population in Bangladesh. Environ. Res..

[60] Gardner R.M., Nermell B., Kippler M., Grandér M., Li L., Ekström E.-C., Rahman A., Lönnerdal B., Hoque A.M.W., Vahter M. (2011). Arsenic methylation efficiency increases during the first trimester of pregnancy independent of folate status. Reprod. Toxicol..

[61] Gardner R.M., Engström K., Bottai M., Hoque W.A.M., Raqib R., Broberg K., Vahter M. (2012). Pregnancy and the methyltransferase genotype independently influence the arsenic methylation phenotype. Pharmacogenet. Genom..

[62] Laine J.E., Bailey K.A., Rubio-Andrade M., Olshan A.F., Smeester L., Drobná Z., Herring A.H., Stýblo M., García-Vargas G.G., Fry R.C. (2015). Maternal arsenic exposure, arsenic methylation efficiency, and birth outcomes in the Biomarkers of Exposure to ARsenic (BEAR) pregnancy cohort in Mexico. Environ. Health Perspect..

[63] Heck J.E., Gamble M.V., Chen Y., Graziano J.H., Slavkovich V., Parvez F., Baron J.A., Howe G.R., Ahsan H. (2007). Consumption of folate-related nutrients and metabolism of arsenic in Bangladesh. Am. J. Clin. Nutr..

[64] Melkonian S., Argos M., Chen Y., Parvez F., Pierce B., Ahmed A., Islam T., Ahsan H. (2012). Intakes of several nutrients are associated with incidence of arsenic-related keratotic skin lesions in Bangladesh. J. Nutr..

[65] Van Geen A., Zheng Y., Cheng Z., He Y., Dhar R.K., Garnier J.M., Rose J., Seddique A., Hoque M.A., Ahmed K.M. (2006). Impact of irrigating rice paddies with groundwater containing arsenic in Bangladesh. Sci. Total Environ..

[66] Williams P.N., Islam M.R., Adomako E.E., Raab A., Hossain S.A., Zhu Y.G., Feldmann J., Meharg A.A. (2006). Increase in Rice Grain Arsenic for Regions of Bangladesh Irrigating Paddies with Elevated Arsenic in Groundwaters. Environ. Sci. Technol..

[67] Nriagu J.O., Lin T.-S. (1995). Trace metals in wild rice sold in the United States. Sci. Total Environ..

[68] Williams P.N., Price A.H., Raab A., Hossain S.A., Feldmann J., Meharg A.A. (2005). Variation in Arsenic Speciation and Concentration in Paddy Rice Related to Dietary Exposure. Environ. Sci. Technol..

[69] Williams P.N., Raab A., Feldmann J., Meharg A.A. (2007). Market Basket Survey Shows Elevated Levels of As in South Central USA Processed Rice Compared to California: Consequences for Human Dietary Exposure. Environ. Sci. Technol..

[70] Basu A., Mitra S., Chung J., Guha Mazumder D.N., Ghosh N., Kalman D., von Ehrenstein O.S., Steinmaus C., Liaw J., Smith A.H. (2011). Creatinine, Diet, Micronutrients, and Arsenic Methylation in West Bengal, India. Environ. Health Perspect..

[71] Laparra J.M., Vélez D., Barberá R., Farré R., Montoro R. (2005). Bioavailability of Inorganic Arsenic in Cooked Rice: Practical Aspects for Human Health Risk Assessments. J. Agric. Food Chem..

[72] He Y., Zheng Y. (2010). Assessment of in vivo bioaccessibility of arsenic in dietary rice by a mass balance approach. Sci. Total Environ..

[73] Davis M.A., Mackenzie T.A., Cottingham K.L., Gilbert-Diamond D., Punshon T., Karagas M.R. (2012). Rice Consumption and Urinary Arsenic Concentrations in USA Children. Environ. Health Perspect..

[74] Jean J.-S., Bundschuh ‎J., Bhattacharya ‎P. (2010). Arsenic in Geosphere and Human Diseases, (Arsenic 2010). Proceedings of the 3rd International Congress on Arsenic in the Environment (As-2010), Tainan, Taiwan, 17–21 May 2010.

[75] Rehman Z.U., Khan S., Qin K., Brusseau M.L., Shah M.T., Din I. (2016). Quantification of inorganic arsenic exposure and cancer risk via consumption of vegetables in southern selected districts of Pakistan. Sci. Total Environ..

[76] Xue J., Zartarian V., Wang S.-W., Liu S.V., Georgopoulos P. (2009). Probabilistic Modeling of Dietary Arsenic Exposure and Dose and Evaluation with 2003–2004 NHANES Data. Environ. Health Perspect..

[77] Awata H., Linder S., Mitchell L.E., Delclos G.L. (2017). Association of Dietary Intake and Biomarker Levels of Arsenic, Cadmium, Lead, and Mercury among Asian Populations in the USA: NHANES 2011–2012. Environ. Health Perspect..

[78] Gagnon F., Lampron-Goulet E., Normandin L., Langlois M.-F. (2016). Measurements of Arsenic in the Urine and Nails of Individuals Exposed to Low Concentrations of Arsenic in Drinking Water From Private Wells in a Rural Region of Québec, Canada. J. Environ. Health.

[79] Molin M., Ulven S.M., Meltzer H.M., Alexander J. (2015). Arsenic in the human food chain, biotransformation and toxicology—Review focusing on seafood arsenic. J. Trace Elem. Med. Biol..

[80] Slotnick M.J., Meliker J.R., Kannan S., Nriagu J.O. (2008). Effects of nutritional measures on toenail arsenic concentration as a biomarker of arsenic exposure. Biomarkers.

[81] Gruber J.F., Karagas M.R., Gilbert-Diamond D., Bagley P.J., Zens M.S., Sayarath V., Punshon T., Morris J.S., Cottingham K.L. (2012). Associations between toenail arsenic concentration and dietary factors in a New Hampshire population. Nutr. J..

[82] Alava P., Du Laing G., Tack F., De Ryck T., Van De Wiele T. (2015). Westernized diets lower arsenic gastrointestinal bioaccessibility but increase microbial arsenic speciation changes in the colon. Chemosphere.

[83] Rahman M.M., Rahman F., Sansom L., Naidu R., Schmidt O. (2009). Arsenic interactions with lipid particles containing iron. Environ. Geochem. Health.

[84] Willet W. (2013). Food Frequency Methods. Nutritional Epidemiology.

[85] World Bank (2011). Bangladesh—Household Income and Expenditure Survey: Key Findings and Results 2010.

[86] Willett W. (2012). Nutritional Epidemiology: CH 13 Issues in Analysis and Presentation of Dietary Data.

[87] Draper N.R., Smith H. (1998). Applied Regression Analysis.

