# Supplementary material for: Associations between Diet and Toenail Arsenic Concentration among Pregnant Women in Bangladesh: A Prospective Study
Source: Nutrients. 2017 Apr 23;9(4):420. doi: 10.3390/nu9040420 (PMC5409759; doi:10.3390/nu9040420)
Supplement: Supplementary file 1 [file nutrients-09-00420-s001.docx]

**Table S1.** Associations between food intake and toenail arsenic concentration using linear regression for the 1st dietary assessment.

| Food Items | Water [As] ≤ 50 ug L^–1^ (*n* = 629) | | |  | Water [As] > 50 ug L^–1^ (*n* = 171) | | |
| --- | --- | --- | --- | --- | --- | --- | --- |
|  | **Crude** | **Adjusted** | **% Change** |  | **Crude** | **Adjusted** | **% Change** |
|  | $\hat{\boldsymbol{\beta}}\left( \mathbf{SE} \right)$ | $\hat{\boldsymbol{\beta}}\left( \mathbf{SE} \right)$ |  |  | $\hat{\boldsymbol{\beta}}\boldsymbol{(SE)}$ | $\hat{\boldsymbol{\beta}}\boldsymbol{(SE)}$ |  |
| Grain, Cereal, Bread | | | | | | | |
| Plain rice *(Bhaat, Panta bhaat)* | 0.453(0.205) | 0.445(0.213) | 13.65 |  | 0.230(0.681) | 0.027(0.714) | 0.79 |
| Special rice *(Khichuri, Pulao, Biriyani)* | 0.042(0.056) | 0.045(0.057) | 6.47 |  | –0.169(0.109) | –0.150(0.111) | –26.23 |
| Rice cereal *(Chira, Muri, Khoi, Murki)* | –0.054(0.025) | –0.050(0.025) | –16.40 |  | –0.297(0.049)** | –0.284(0.051)** | –62.00 |
| Plain bread *(Atta ruti, Pau ruti)* | 0.030(0.032) | 0.025(0.035) | 6.28 |  | 0.028(0.066) | 0.027(0.071) | 7.73 |
| Fried bread *(Porota, Luchi)* | –0.007(0.050) | –0.018(0.055) | –3.46 |  | –0.673(0.154)** | –0.679(0.167)** | –61.80 |
| Homemade snacks *(Pitha–puli)* | –0.075(0.039) | –0.064(0.041) | –15.21 |  | –0.398(0.076)** | –0.4(000.076)** | –61.77 |
| Vegetable | | | | | | | |
| Leafy vegetable *(Sak)* | 0.055(0.051) | 0.044(0.053) | 6.29 |  | 0.439(0.106)** | 0.447(0.109)** | 85.95 |
| Mashed vegetable *(Bhorta)* | 0.045(0.031) | 0.045(0.032) | 13.05 |  | 0.147(0.066)* | 0.172(0.067)** | 59.42 |
| Fried vegetable *(Bhaji)* | 0.057(0.039) | 0.052(0.040) | 15.33 |  | 0.210(0.084)** | 0.250(0.089)** | 97.20 |
| Mixed vegetable *(Labra)* | –0.009(0.035) | –0.014(0.036) | –4.79 |  | –0.141(0.065)* | –0.148(0.068)** | –39.72 |
| Vegetable curry (Torkarir jhole) | 0.004(0.042) | 0.011(0.043) | 3.13 |  | –0.183(0.067)** | –0.178(0.069)** | –45.60 |
| Legumes, Pulses, Seed | | | | | | | |
| Plain dal | –0.059(0.039) | –0.050(0.039) | –12.83 |  | –0.105(0.072) | –0.146(0.076)* | –39.28 |
| Dal with vegetables | –0.051(0.082) | –0.001(0.088) | –0.11 |  | –0.474(0.195)** | –0.608(0.203)** | –49.19 |
| Fish, Poultry, Meat, Egg | | | | | | | |
| Fish fry *(Mach bhaji)* | –0.068(0.032) | –0.066(0.032) | –18.61 |  | –0.285(0.063)** | –0.286(0.065)** | –54.10 |
| Fish curry *(Mach er jhole)* | 0.087(0.034) | 0.092(0.036) | 36.93 |  | 0.368(0.104)** | 0.382(0.115)** | 116.90 |
| Fish curry with vegetable | 0.070(0.034) | 0.071(0.036) | 21.21 |  | 0.504(0.108)** | 0.524(0.111)** | 189.10 |
| Fish head with dal or vegetables | –0.043(0.046) | –0.029(0.047) | –5.62 |  | –0.561(0.101)** | –0.574(0.101)** | –74.44 |
| Fish egg fry (Maccher dim bhaji) | –0.039(0.033) | –0.022(0.035) | –6.83 |  | –0.507(0.075)** | –0.530(0.077)** | –77.12 |
| Dried fish with vegetable | 0.006(0.024) | –0.002(0.026) | –1.01 |  | 0.135(0.058)* | 0.149(0.062)* | 64.23 |
| Meat curry with potato | 0.038(0.039) | 0.033(0.041) | 9.51 |  | 0.300(0.147)* | 0.271(0.159) | 20.64 |
| Meat with legumes *(Halim)* | –0.201(0.107) | –0.154(0.147) | –13.98 |  | 0.338(0.241) | 0.030(0.335) | 2.98 |
| Meat with grains, legumes, vegetables (*Dhansak*) | - | - | - |  | - | - | - |
| Meat kebab | 0.002(0.021) | 0.017(0.057) | 9.20 |  | 0.085(0.048)* | 0.771(0.122)** | 3321.24 |
| Egg curry *(Dim er jhole)* | –0.016(0.033) | –0.022(0.034) | –7.24 |  | 0.113(0.069)* | 0.134(0.080) | 43.85 |
| Milk | | | | | | | |
| Plain milk *(Doodh)* | –0.040(0.029) | –0.039(0.030) | –13.78 |  | –0.073(0.064) | –0.102(0.069) | –32.26 |
| Cottage cheese *(chana)* | –0.104(0.065) | –0.074(0.075) | –11.58 |  | –0.874(0.144)** | –1.053(0.150)** | –79.78 |
| Yogurt *(Doi)* | –0.089(0.047) | –0.077(0.049) | –13.87 |  | –0.636(0.090)** | –0.618(0.092)** | –71.94 |
| Yogurt drink *(Ghole, Matha, Borhani)* | –0.043(0.048) | –0.114(0.077) | –21.63 |  | –0.003(0.116) | –0.307(0.176) | –44.92 |
| Thickened milk *(Khoa, kheer)* | –0.096(0.079) | –0.078(0.082) | –10.20 |  | –0.245(0.134)* | –0.236(0.138) | –27.50 |
| Rice pudding *(Payesh)* | –0.024(0.048) | –0.014(0.049) | –2.96 |  | –0.581(0.108)** | –0.577(0.112)** | –68.47 |
| Vermicelli *(Semai)* | –0.049(0.045) | –0.039(0.046) | –9.81 |  | –0.182(0.116) | –0.192(0.120) | –23.35 |
| Sweetmeats (*Mishti*) | - | - | - |  | - | - | - |
| Fruits | | | | | | | |
| Fruit | –0.070(0.029) | –0.063(0.031) | –17.87 |  | –0.300(0.062)** | –0.316(0.063)** | –58.55 |
| Mashed fruit *(Bhorta)* | –0.002(0.074) | 0.057(0.089) | 7.93 |  | –0.217(0.143) | –0.519(0.163)** | –49.13 |
| Fruit pickle *(Aachar)* | –0.116(0.046) | –0.111(0.046) | –24.05 |  | –0.465(0.085)** | –0.457(0.086)** | –78.20 |
| Beverages | | | | | | | |
| Fruit juice | –0.081(0.042) | –0.099(0.043) | –21.87 |  | –0.548(0.089)** | –0.612(0.095)** | –76.25 |
| Soft drinks | –0.104(0.061) | –0.097(0.067) | –15.11 |  | –0.809(0.145)** | –0.861(0.149)** | –75.45 |
| Tea | –0.067(0.044) | –0.043(0.046) | –9.65 |  | –0.602(0.094)** | –0.619(0.095)** | –77.18 |
| Coffee | - | - | - |  | - | - | - |

“Crude” indicates a model adjusted for water arsenic concentration only. “Adjusted” indicates a model with adjustment for water arsenic level, sex, smoking in the living environment, chewing betel nut, BMI, daily water intake, daily energy intake, and education level. Units for the estimated coefficients are natural-log transformed ((toenail As concentrations, μg/g)·(g/d)^−1^). Q-value accounting for multiple comparisons using the false discovery rate (FDR = 0.05) method (** *Q*-value < 0.01; * *Q*-value < 0.05).

**Table S2.** Associations between food intake and toenail arsenic concentrations using linear regression for the 2nd dietary assessment.

| Food Items | Water [As] ≤50 ug L^–1^ (*n* = 683) | | |  | Water [As] > 50 ug L^–1^ (*n* = 208) | | | |
| --- | --- | --- | --- | --- | --- | --- | --- | --- |
|  | **Crude** | **Adjusted** | **% change** |  | **Crude** | **Adjusted** | | **% change** |
|  | $\hat{\boldsymbol{\beta}}\boldsymbol{(SE)}$ | $\hat{\boldsymbol{\beta}}\boldsymbol{(SE)}$ |  |  | $\hat{\boldsymbol{\beta}}\boldsymbol{(SE)}$ | $\hat{\boldsymbol{\beta}}\boldsymbol{(SE)}$ | |  |
| Grain, Cereal, Bread | | | |  |  |  | |  |
| Plain rice *(Bhaat, Panta bhaat)* | 0.146(0.258) | 0.119(0.258) | 4.95 |  | –0.850(0.701) | –0.935(0.722) | | –3.22 |
| Special rice *(Khichuri, Pulao, Biriyani)* | 0.012(0.066) | –0.003(0.066) | –0.48 |  | 0.046(0.081) | 0.068(0.089) | | 9.89 |
| Rice cereal *(Chira, Muri, Khoi, Murki)* | –0.077(0.025)** | –0.071(0.026)* | –21.67 |  | –0.042(0.053) | –0.032(0.054) | | –8.43 |
| Plain bread *(Atta ruti, Pau ruti)* | 0.015(0.038) | –0.007(0.039) | –1.84 |  | –0.040(0.054) | –0.071(0.062) | | –13.32 |
| Fried bread *(Porota, Luchi)* | –0.179(0.047)** | –0.148(0.050)** | –27.46 |  | 0.040(0.122) | 0.064(0.136) | | 8.76 |
| Homemade snacks *(Pitha–puli)* | –0.207(0.042)** | –0.186(0.043)** | –37.83 |  | –0.141(0.079) | –0.120(0.083) | | –21.34 |
| Vegetable | | | |  |  |  | |  |
| Leafy vegetable *(Sak)* | 0.254(0.054)** | 0.224(0.057)** | 36.43 |  | 0.103(0.102) | 0.129(0.110) | | 9.35 |
| Mashed vegetable *(Bhorta)* | 0.059(0.031) | 0.044(0.032) | 12.59 |  | 0.083(0.050) | 0.098(0.053) | | 30.41 |
| Fried vegetable *(Bhaji)* | 0.125(0.046)** | 0.091(0.048) | 27.93 |  | 0.061(0.066) | 0.092(0.074) | | 28.30 |
| Mixed vegetable *(Labra)* | 0.023(0.035) | 0.000(0.035) | –0.10 |  | 0.075(0.047) | 0.083(0.049) | | 31.97 |
| Vegetable curry (Torkarir jhole) | –0.031(0.043) | –0.024(0.043) | –4.94 |  | 0.085(0.052) | 0.095(0.054) | | 38.48 |
| Legumes, Pulses, Seed | | | |  |  |  | |  |
| Plain dal | –0.067(0.049) | –0.063(0.050) | –12.07 |  | –0.087(0.055) | –0.085(0.058) | | –25.19 |
| Dal with vegetables | –0.132(0.030)** | –0.113(0.031)** | –28.83 |  | –0.087(0.099) | –0.137(0.104) | | –12.57 |
| Fish, Poultry, Meat, Egg | | | |  |  |  | |  |
| Fish Fry *(Mach bhaji)* | –0.131(0.030)** | –0.118(0.032)** | –30.76 |  | –0.042(0.063) | –0.005(0.068) | | –1.26 |
| Fish curry *(Mach er jhole)* | 0.139(0.035)** | 0.113(0.037)** | 47.27 |  | 0.044(0.083) | 0.081(0.086) | | 17.76 |
| Fish curry with vegetable | 0.135(0.036)** | 0.105(0.037)* | 24.30 |  | 0.247(0.119) | 0.336(0.133) | | 26.18 |
| Fish head with dal or vegetables | –0.215(0.052)** | –0.187(0.053)** | –28.62 |  | 0.019(0.101) | 0.063(0.106) | | 13.42 |
| Fish egg fry (Maccher dim bhaji) | –0.138(0.028)** | –0.122(0.03)** | –35.29 |  | –0.024(0.087) | 0.001(0.090) | | 0.26 |
| Dried fish with vegetable | 0.098(0.023)** | 0.079(0.025)** | 38.37 |  | 0.042(0.051) | 0.047(0.064) | | 13.71 |
| Meat curry with potato | 0.168(0.047)** | 0.127(0.048)* | 41.15 |  | –0.183(0.126) | –0.077(0.139) | | –5.22 |
| Meat with legumes *(Halim)* | 0.064(0.041) | 0.101(0.088) | 32.98 |  | 0.014(0.072) | 0.285(0.177) | | 102.25 |
| Meat with grains, legumes, vegetables (*Dhansak*) | - | - | - |  | - | - | | - |
| Meat kebab | 0.107(0.058) | 0.222(0.112) | 54.79 |  | 0.160(0.093) | 0.179(0.232) | | 37.86 |
| Egg curry *(Dim er jhole)* | –0.001(0.043) | –0.032(0.044) | –8.45 |  | –0.048(0.053) | –0.012(0.064) | | –3.31 |
| Milk | | | |  |  |  | |  |
| Plain milk *(Doodh)* | –0.065(0.037) | –0.058(0.038) | –13.33 |  | 0.057(0.049) | 0.081(0.052) | | 36.04 |
| Cottage cheese *(chana)* | –0.134(0.033)** | –0.132(0.038)** | –32.93 |  | –0.233(0.104) | –0.255(0.155) | | –28.02 |
| Yogurt *(Doi)* | –0.179(0.049)** | –0.152(0.05)** | –25.05 |  | –0.136(0.135) | | –0.080(0.142) | –9.96 |
| Yogurt drink *(Ghole, Matha, Borhani)* | - | - | - |  | - | | - | - |
| Thickened milk *(Khoa, kheer)* | –0.157(0.081) | –0.142(0.086) | –18.26 |  | –0.099(0.092) | | –0.063(0.095) | –15.66 |
| Rice pudding *(Payesh)* | –0.203(0.047)** | –0.175(0.048)** | –32.05 |  | –0.053(0.096) | | –0.077(0.100) | –9.59 |
| Vermicelli *(Semai)* | –0.093(0.041)* | –0.075(0.042) | –18.49 |  | 0.000(0.092) | | 0.008(0.096) | 1.16 |
| Sweetmeats (*Mishti*) | - | - | - |  | - | | - | - |
| Fruits | | | |  |  | |  |  |
| Fruit | –0.162(0.031)** | –0.154(0.032)** | –38.10 |  | –0.088(0.050) | | –0.054(0.054) | –13.78 |
| Mashed fruit *(Bhorta)* | 0.059(0.028)* | 0.086(0.044) | 41.70 |  | 0.013(0.053) | | 0.189(0.091) | 77.70 |
| Fruit pickle *(Aachar)* | –0.184(0.040)** | –0.170(0.042)** | –37.58 |  | –0.108(0.105) | | –0.085(0.110) | –15.64 |
| Beverages | | | |  |  | |  |  |
| Fruit juice | 0.003(0.015) | 0.019(0.045) | 15.73 |  | 0.003(0.025) | | 0.031(0.085) | 21.76 |
| Soft drinks | –0.205(0.055)** | –0.188(0.058)** | –28.95 |  | –0.183(0.182) | | –0.274(0.204) | –20.71 |
| Tea | –0.141(0.038)** | –0.124(0.039)** | –26.73 |  | –0.164(0.138) | | –0.226(0.146) | –20.03 |
| Coffee | - | - | - |  | - | | - | - |

“Crude” indicates a model adjusted for water arsenic concentration only. “Adjusted” indicates a model with adjustment for water arsenic level, sex, smoking in the living environment, chewing betel nut, BMI, daily water intake, daily energy intake, and education level. Units for the estimated coefficients are natural-log transformed ((toenail As concentrations, μg/g)·(g/d)^−1^). “As-adjusted” indicates a model adjusted for water arsenic concentration only. “Fully adjusted” indicates a model with adjustment for water arsenic level, sex, smoking in the living environment, chewing betel nut, BMI, daily water intake, daily energy intake, and education level.
